# Prevalence of Clinical Signs Within Reference Ranges Among Hospitalized Patients Prescribed Antibiotics for Pneumonia

**DOI:** 10.1001/jamanetworkopen.2020.10700

**Published:** 2020-07-17

**Authors:** Michael Klompas, Aileen Ochoa, Wenjing Ji, Caroline McKenna, Roger Clark, Erica S. Shenoy, David Hooper, Chanu Rhee

**Affiliations:** 1 Harvard Pilgrim Health Care Institute, Department of Population Medicine, Harvard Medical School, Boston, Massachusetts; 2 Department of Medicine, Brigham and Women’s Hospital, Boston, Massachusetts; 3 Department of Pharmacy Administration and Clinical Pharmacy, Xi’an Jiaotong University School of Pharmacy, Xi’an, Shaanxi, China; 4 Department of Medicine, Brigham and Women’s Faulkner Hospital, Boston, Massachusetts; 5 Infection Control Unit, Massachusetts General Hospital, Boston, Massachusetts

## Abstract

**Importance:**

Antibiotics are frequently prescribed for suspected pneumonia, but overdiagnosis is common and fixed regimens are often used despite randomized trials suggesting it is safe to stop antibiotics once clinical signs are normalizing.

**Objective:**

To quantify potential excess antibiotic prescribing by characterizing antibiotic use relative to patients’ initial clinical signs and subsequent trajectories.

**Design, Setting, and Participants:**

An observational cohort study was conducted in 2 tertiary and 2 community hospitals in Eastern Massachusetts. All nonventilated adult patients admitted between May 1, 2017, and July 1, 2018 (194 521 hospitalizations), were included.

**Main Outcomes and Measures:**

Identification of all antibiotic starts for possible community-acquired pneumonia (CAP) or hospital-acquired pneumonia (HAP) per clinicians’ stated indications. Potential excess antibiotic prescribing was quantified by characterizing the frequency of patients in whom all clinical signs were within reference ranges on the first day of antibiotic therapy and by how long antibiotic therapy was continued after all clinical signs were normal, including postdischarge antibiotics.

**Results:**

Among 194 521 hospitalizations, 9540 patients were treated for possible CAP (4574 [48.0%] women; mean [SD] age, 67.6 [17.0] years) and 2733 for possible HAP (1211 [44.3%] women; mean [SD] age, 66.7 [16.2] years). Temperature, respiratory rate, oxygen saturation, and white blood cell count were all within reference ranges on the first day of antibiotics in 1779 of 9540 (18.6%) episodes of CAP and 370 of 2733 (13.5%) episodes of HAP. Antibiotics were continued for 3 days or longer after all clinical signs were normal in 3322 of 9540 (34.8%) episodes of CAP and 1050 of 2733 (38.4%) episodes of HAP. Up to 24 978 of 71 706 (34.8%) antibiotic-days prescribed for possible pneumonia may have been unnecessary.

**Conclusions and Relevance:**

In this study, almost one-fifth of hospitalized patients treated for pneumonia did not have any of the cardinal signs of pneumonia on the first day of treatment and antibiotics were continued for 3 days or longer after all signs were normal in more than a third of patients. These observations suggest substantial opportunities to improve antibiotic prescribing.

## Key Points

**Question:**

What is the prevalence of antibiotic therapy for possible pneumonia in hospitalized patients despite clinical signs within the reference range?

**Findings:**

In this cohort study of 12 273 patients treated for possible pneumonia in 4 hospitals, all cardinal signs for pneumonia were within reference ranges in 18.6% of patients with possible community-acquired pneumonia and 13.5% of patients with possible hospital-acquired pneumonia. Antibiotics were continued for 3 days or longer after all clinical signs were normal in 34.8% of patients treated for community-acquired pneumonia and 38.4% treated for hospital-acquired pneumonia.

**Meaning:**

Findings of this study suggest that antibiotics are prescribed frequently for suspected pneumonia in patients with clinical signs within reference ranges and continued for 3 days or longer after clinical signs normalize; these findings suggest potential targets to improve prescribing.

## Teaser

This cohort study examines the prescribing rates and duration of antibiotic therapy in patients with possible pneumonia whose clinical signs are within the reference ranges.

## Introduction

The most common indication for antibiotics in hospitalized patients is suspected respiratory tract infection.^1,2^ Diagnosing pneumonia, however, is difficult. The cardinal signs of pneumonia (fever, impaired oxygenation, tachypnea, productive cough, leukocytosis, and radiographic infiltrates) are not specific for pneumonia, individually or collectively.^3,4,5,6^ Many acute and chronic conditions mimic the clinical presentation of pneumonia, including congestive heart failure, exacerbations of obstructive lung disease, atelectasis, mucus plugging, pulmonary embolism, hypersensitivity reactions, and others. Radiographic infiltrates are frequent in hospitalized patients and difficult to interpret: interobserver variability is high and specificity for pneumonia is low.^7,8,9,10^ Notwithstanding the difficulty of accurately diagnosing pneumonia, it is a common diagnosis and therefore frequently invoked by clinicians to explain patients’ signs and symptoms. The net result of these many potential sources of error is that overdiagnosis of pneumonia is common and may account for a substantial fraction of unnecessary antibiotic use in hospitalized patients.^11,12,13,14^

The problem is often compounded by continuing antibiotic treatment for longer than needed.^15^ Guidelines recommend treating community-acquired pneumonia (CAP) for 5 days and hospital-acquired pneumonia (HAP) for 7 days, but adherence to these guidelines is poor.^16,17,18,19,20^ Moreover, at least 3 randomized clinical trials suggest that it is safe to stop antibiotic treatment once clinical signs are normalizing, even after as few as 3 days.^21,22,23^ We sought to quantify the frequency of potentially unnecessary prescribing for pneumonia by identifying the percentage of hospitalized patients started on antibiotic therapy for possible CAP and HAP with clinical signs within reference ranges on the first day of antibiotic administration, the number of days until clinical signs normalized among patients with at least 1 abnormal clinical sign on the first day of antibiotic treatment, and the duration of therapy beyond when clinical signs normalized.

## Methods

We conducted an observational cohort study of all patients aged 18 years or older admitted to 4 hospitals in the greater Boston, Massachusetts, area between May 1, 2017, and July 1, 2018. Within this cohort, we retrospectively identified nonventilated patients started on antibiotic therapy for possible pneumonia according to the indications provided by clinicians at the time antibiotic regimens were ordered. Study hospitals included 2 academic medical centers (Brigham and Women’s Hospital and Massachusetts General Hospital) and 2 community hospitals (Newton Wellesley Hospital and Faulkner Hospital). We included both CAP (defined as antibiotic treatment started for pneumonia on hospital day 1 or 2) and HAP (antibiotic treatment started on hospital day 3 or thereafter) but analyzed the 2 groups separately. Antibiotic indications were determined from the indications specified by clinicians using a required field within the medication-ordering interface in the hospitals’ electronic health record systems. We included the indication pneumonia selected from the structured set of options offered by the electronic health record, free-text indications added by clinicians that were concordant with pneumonia, and antibiotic courses initially prescribed for a stated indication of sepsis but modified at a later date to explicitly state pneumonia. We excluded patients with cystic fibrosis, empyema, abscess, or other pyogenic complications per discharge *International Statistical Classification of Diseases, 10th Revision* (*ICD-10*) codes E84, J86, and J85. We assessed the validity of our inclusion criteria by randomly selecting 60 eligible patients and reviewing their medical records to determine clinicians’ intentions on the day antibiotic treatment was started (as opposed to their discharge diagnoses) per the diagnostic impression, differential diagnosis, or plan recorded in the free-text clinical notes.

All study hospitals used the Epic Care electronic health record system (Epic Systems). All study hospitals have longstanding antibiotic stewardship programs that include guidance on typical treatment durations for both CAP and HAP but do not provide explicit guidance on how to diagnose pneumonia. The study was approved by the Partners Healthcare Institutional Review Board with a waiver of informed consent because findings are only presented in aggregate. This study followed the Strengthening the Reporting of Observational Studies in Epidemiology (STROBE) reporting guideline for cohort studies.

Patients' comorbidities were derived from their discharge *ICD-10* codes using the Charlson^24^ and Elixhauser^25^ scales. We determined the percentages of patients with clinical signs within the reference ranges on the first calendar day of antibiotic therapy both per sign and collectively across all signs. We defined temperature as normal if the daily maximum temperature was greater than 36 °C and less than 38 °C, respiratory rate as normal if the median daily respiratory rate was less than 22 breaths/min, white blood cell count as normal if greater than 4000/μL and less than 12 000/μL (to convert to ×10^9^/L, multiply by 0.001), and oxygenation as normal if the median daily oxygen saturation was 95% or greater without supplementary oxygen. We used median values for respiratory rates and oxygen saturations to minimize the impact of outlier values due to data entry errors, machine misreadings, or transient fluctuations in clinical status. We then determined mean and median calendar days until normalization for each clinical sign and for all clinical signs. We did not evaluate chest radiographic imaging findings due to their subjectivity, lack of specificity, the lag between acute disease and radiographic resolution, the difficulty reliably parsing free-text narratives electronically, and low likelihood that a patient has pneumonia if their sole abnormality is a radiographic opacity and all other clinical signs are within reference ranges.^7,8,9,10,26^ We excluded patients missing any reports of temperature, respiratory rate, white blood cell count, or oxygen saturation on the first day of antibiotic administration.

### Statistical Analysis

We assessed mean (SD) and median (interquartile range [IQR]) durations of antibiotic treatment among all patients, those with at least 1 abnormal clinical sign on the first day of treatment, and those with all clinical signs within reference ranges on the first day of antibiotic administration. We calculated the duration of antibiotic treatment beyond the first day of clinical signs within reference ranges in each of these 3 groups (starting from the first calendar day of antibiotic administration for patients in whom all signs were within reference ranges and from the first calendar day that all signs normalized for patients with initially abnormal clinical signs). We further assessed the count and percentage of patients who were given 3 or more and 5 or more days of antibiotic therapy beyond when all clinical signs normalized. Discharge antibiotic courses were included in all calculations.

We conducted multiple sensitivity analyses. The first analysis was restricted to patients with discharge *ICD-10* diagnosis codes for pneumonia (J13-J18) in order to focus on a subset of patients in whom clinicians’ had a sustained impression of pneumonia (thus excluding patients whose diagnosis might have evolved from pneumonia to some other infection that merited a longer course of antibiotic treatment). The second sensitivity analysis was restricted to patients with negative results of blood and sputum cultures to exclude antibiotic administration being continued to treat bacteremia or in case clinicians believed that sputum culture–positive disease required longer treatment courses. The third sensitivity analysis excluded immunocompromised patients, defined as all patients admitted to an oncology service, transplant service, or with discharge diagnosis codes for immunodeficiency (*ICD-10* code D80-D84) or an organ transplant (*ICD*-*10* code Z94). The fourth sensitivity analysis added blood pressure to the evaluation of clinical signs on the first calendar day of antibiotic administration and subsequent treatment courses. Hypotension was defined as a minimum systolic blood pressure of 90 mm Hg or lower. Data compilation and analysis were performed using SAS, version 9.4 (SAS Institute Inc).

## Results

Among 194 521 unique admissions during the study period, antibiotic therapy was initiated in 12 273 patients (6.3%; mean [SD] age, 67.4 [16.4] years) for possible pneumonia (5785 women [47.1%]). Of these 12 273 patients, 9540 individuals (77.7%; 4574 [48.0%] women; mean [SD] age, 67.6 [17.0] years) received antibiotics for possible CAP and 2733 patients (22.3%; 1211 [44.3%] women; mean [SD] age, 66.7 [16.2] years) received antibiotics for possible HAP. A total of 71 706 antibiotic-days for treatment of suspected pneumonia (55 609 inpatient antibiotic-days, 16 097 postdischarge antibiotic-days) were noted relative to 723 029 total antibiotic-days for any indication other than prophylaxis (350 679 inpatient antibiotic-days, 372 350 postdischarge antibiotic-days). As such, prescribing for pneumonia accounted for 9.9% of all antibiotic-days and 15.9% of all inpatient antibiotic-days.

Characteristics of patients who received antibiotics for possible CAP and HAP are summarized in Table 1. Clinicians’ specified indications for antibiotic treatment were consistent with the clinical impressions documented in clinical notes: suspicion for pneumonia on the day antibiotic administration was started was confirmed in 56 of 60 medical records randomly selected for review (positive predictive value, 93%; 95% CI, 85%-98%). The 4 false-positive findings included 2 patients prescribed antibiotic therapy for chronic obstructive lung disease exacerbations, 1 patient with acute retropharyngeal inflammation of unclear cause, and 1 patient who received a lung transplant and was prescribed azithromycin and trimethoprim with sulfamethoxazole for prophylaxis against opportunistic infections.

**Table 1.** Patient Characteristics

Characteristic	Pneumonia, No. (%)	
	Community-acquired (n = 9540)	Hospital-acquired (n = 2733)
Demographics		
Age, mean (SD), y	67.6 (17.0)	66.7 (16.2)
Women	4574 (48.0)	1211 (44.3)
Race/ethnicity		
White	7500 (78.6)	2230 (81.6)
Black	833 (8.7)	204 (7.5)
Hispanic	327 (3.4)	64 (2.3)
Asian	308 (3.2)	94 (3.4)
Other	572 (6.0)	141 (5.2)
Clinical service on day 1 of antibiotics for pneumonia		
Cardiac surgery	13 (0.1)	38 (1.4)
Cardiology	190 (2.0)	80 (2.9)
Emergency^a^	2450 (25.7)	32 (1.2)
Gynecology	4 (0.0)	7 (0.3)
Intensive care	634 (6.7)	346 (12.7)
Medicine	4641 (48.7)	1093 (40.0)
Neurology	88 (0.9)	120 (4.4)
Obstetrics	11 (0.1)	8 (0.3)
Oncology	1262 (13.2)	626 (22.9)
Surgery	242 (2.5)	378 (13.8)
Other	5 (0.1)	5 (0.2)
Comorbidities^b^		
Congestive heart failure	1787 (18.7)	534 (19.5)
Myocardial infarction	468 (4.9)	154 (5.6)
Chronic pulmonary disease	2413 (25.3)	407 (14.9)
Dementia	392 (4.1)	94 (3.4)
Diabetes	1629 (17.1)	451 (16.6)
Liver disease	370 (3.9)	166 (6.1)
Neurologic disease	423 (4.4)	308 (13.3)
Chronic kidney disease	1111 (11.6)	281 (10.3)
Cancer	1696 (17.8)	672 (24.6)
Charlson comorbidity index score, mean (SD)	2.0 (2.2)	2.3 (2.3)
Outcomes		
Length of stay, median (IQR)	5 (3-9)	13 (8-23)
Hospital death	610 (6.4)	421 (15.4)

Abbreviation: IQR, interquartile range.

^a^ Discharge services for patients initially prescribed antibiotics for pneumonia in the emergency department included medicine (51.3%), emergency department observation unit (36.3%), oncology (7.4%), and surgery (2.6%).

^b^ Comorbidities derived from discharge diagnosis codes using the methods of Charlson^24^ and Elixhauser.^25^

Percentages of patients with clinical signs within reference ranges on the first day of antibiotic administration are summarized in Table 2. Among patients who started receiving antibiotics for possible CAP, per our definitions (ie, according to clinicians’-stated indications), 7499 individuals (78.6%) had normal temperatures, 7779 patients (81.5%) had normal median respiratory rates, 5253 patients (55.1%) had normal white blood cell counts, and 3717 patients (39.0%) had median oxygen saturations greater than or equal to 95% without supplementary oxygen. Among those who began receiving antibiotics for possible HAP, per our definitions, 1935 patients (70.8%) had normal temperatures, 2182 patients (79.8%) had normal respiratory rates, 1326 patients (48.5%) had normal white blood cell counts, and 955 patients (34.9%) had median oxygen saturations greater than or equal to 95% without supplementary oxygen. All clinical signs were within reference ranges in 1799 patients (18.7%) with possible CAP and 370 patients (13.5%) with possible HAP. Among patients with at least 1 abnormal clinical sign on the first day of antibiotic therapy (7268 treated for CAP, 2200 treated for HAP), all clinical signs normalized within a median of 3 days (IQR, 2-6) for possible CAP and 4 days (IQR, 2-9) for possible HAP.

**Table 2.** Clinical Signs on the First Day of Antibiotic Therapy

Variable	Pneumonia, No. (%)	
	Community-acquired (n = 9540)	Hospital-acquired (n = 2733)
Frequency of signs normal on day 1 of antibiotic therapy		
Daily maximum temperature >36 °C and <38 °C	7499 (78.6)	1935 (70.8)
Median daily respiratory rate ≤22 breaths/min	7779 (81.5)	2182 (79.8)
Daily maximum white blood cell count >4000/μL and <12 000/μL	5253 (55.1)	1326 (48.5)
Not on supplemental oxygen	5028 (52.7)	1468 (53.7)
Oxygen saturation ≥95% without supplemental oxygen	3717 (39.0)	955 (34.9)
Median respiratory rate ≤22 breaths/min and oxygen saturation ≥95% without supplemental oxygen	3506 (36.8)	877 (32.1)
All signs within reference ranges	1779 (18.6)	370 (13.5)
**Days until clinical signs normal for patients with**≥**1 abnormal sign on day 1 of antibiotic therapy**		
Temperature		
Mean (SD)	1.6 (1.3)	2.0 (1.9)
Median (IQR)	1 (1-2)	1 (1-2)
White blood cell count		
Mean (SD)	3.7 (4.1)	5.7 (6.5)
Median (IQR)	2 (1-4)	3 (2-7)
Median respiratory rate		
Mean (SD)	2.2 (2.2)	2.6 (3.0)
Median (IQR)	1 (1-3)	2 (1-3)
Off supplemental oxygen		
Mean (SD)	4.1 (4.1)	3.8 (4.1)
Median (IQR)	3 (1-6)	2 (1-5)
Off supplemental oxygen and saturation ≥95%		
Mean (SD)	4.5 (5.4)	5.8 (8.2)
Median (IQR)	3 (1-6)	3 (1-7)
Median respiratory rate <22 breaths/min and oxygen saturation ≥95%		
Mean (SD)	3.5 (4.2)	4.9 (5.5)
Median (IQR)	2 (1-4)	3 (1-6)
All signs normal		
Mean (SD)	5.0 (5.7)	7.2 (8.8)
Median (IQR)	3 (2-6)	4 (2-9)

Abbreviation: IQR, interquartile range.

SI conversion: To convert white blood cell count to ×10^9^/L, multiply by 0.001.

Duration of antibiotic therapy is reported in Table 3. Antibiotics were prescribed for a median of 5 days (IQR, 2-7) for CAP but for 7 days or longer in 2951 of 9540 patients with CAP (30.9%). Antibiotics were also prescribed for a median of 5 days for HAP (IQR, 3-8) but for 10 days or longer in 475 of 2733 patients with HAP (17.4%).

**Table 3.** Duration of Antibiotics Relative to Clinical Signs

Variable	Pneumonia	
	Community-acquired (n = 9540)	Hospital-acquired (n = 2733)
Total duration of treatment, d		
All patients		
Mean (SD)	5.6 (5.6)	6.8 (7.2)
Median (IQR)	5 (2-7)	5 (3-8)
Patients with normal signs on 1st day of antibiotics		
Mean (SD)	4.6 (5.8)	5.5 (5.9)
Median (IQR)	3 (1-6)	5 (2-7)
Patients with abnormal signs on 1st day of antibiotics		
Mean (SD)	5.8 (5.5)	7.0 (7.3)
Median (IQR)	5 (2-7)	6 (3-8)
**Duration of treatment beyond the last day of abnormal signs**		
All patients		
Mean (SD)	2.5 (4.5)	3.2 (6.2)
Median (IQR)	1 (0-4)	1 (0-5)
Patients with abnormal signs on day 1 of antibiotics		
Mean (SD)	2.3 (4.3)	2.4 (5.7)
Median (IQR)	0 (0-4)	0 (0-4)
**Patients with extended courses of antibiotics, No. (%)**		
Antibiotics beyond the last day of abnormal signs		
≥3 d	3322 (34.8)	1050 (38.4)
≥5 d	1887 (19.8)	708 (25.9)

Abbreviation: IQR, interquartile range.

Among patients with clinical signs within reference ranges on the first day of antibiotic therapy, the treatment was continued for a median of 4 days (IQR, 1-6) for possible CAP and 5 days (IQR, 3-7) for possible HAP. Among patients with signs outside the reference range on the first day of antibiotic therapy, the treatment was continued for a median of 0 days (IQR, 0-4) beyond when all clinical signs normalized for both possible CAP and possible HAP. Antibiotic therapy was continued for 3 days or longer and 5 days or longer beyond the time when all clinical signs normalized among 3322 patients (34.8%) and 1887 patients (19.8%), respectively, of 9540 individuals with possible CAP and 1050 patients (38.4%) and 708 patients (25.9%), respectively, of 2733 individuals with possible HAP. Across all 4 hospitals, of 71 706 inpatient and discharge days of antibiotic treatment prescribed for possible pneumonia, 10 254 antibiotic-days were documented for patients in whom all clinical signs were within reference ranges on the first day of antibiotic administration and 14 724 antibiotic-days were prescribed counting from day 3 after all clinical signs had normalized among patients with initially abnormal signs.. These 2 figures suggest that as many as 24 978 of 71 706 antibiotic-days (34.8%) of treatment prescribed for possible pneumonia may have been unnecessary.

Results stratified by hospital are presented in eTable 1 and eTable 2 in the Supplement. All findings were broadly consistent across all hospitals. Sensitivity analyses are presented in eTables 3, 4, 5, and 6 in the Supplement. Discharge diagnosis codes for pneumonia were assigned to 3228 of the 9450 patients (34.1%) started on antibiotic therapy for possible CAP and 530 of the 2733 patients (19.4%) administered antibiotics for possible HAP.

Frequencies of clinical signs within reference ranges, overall treatment durations, and duration of treatment beyond the time that clinical signs normalized were generally similar in all sensitivity analyses, albeit slightly longer among patients with discharge diagnosis codes for pneumonia (eTable 3 and eTable 4 in the Supplement) and among patients with negative blood and sputum culture results (eTable 3 and eTable 4 in the Supplement), and slightly shorter among patients who were not immunocompromised (eTable 5 and eTable 6 in the Supplement). Findings were also similar when incorporating blood pressure into the analysis of clinical signs on the first day of antibiotic treatment: 8441 (88.5%) and 2456 (89.9%) patients treated for possible CAP and HAP had systolic blood pressures greater than 90 mm Hg; all clinical signs, including blood pressure, were within the reference range in 2135 patients (22.4%) with CAP and 498 patients (18.2%) with HAP; 3288 patients (34.5%) with CAP and 1032 patients (37.8%) with HAP were treated for 3 days or more beyond when all clinical signs normalized; and 1856 patients (19.5%) with CAP and 692 patients (25.3%) with HAP were treated for 5 days or more beyond when all clinical signs normalized.

## Discussion

Using detailed electronic health record data from 4 hospitals, we found that all the cardinal clinical signs typically associated with pneumonia were within reference ranges among 18.7% of hospitalized patients treated for possible CAP and 13.5% of patients treated for possible HAP. Antibiotic therapy was continued for 3 days or more beyond when clinical signs normalized in 34.8% of patients with possible CAP and 38.4% of patients with possible HAP. Many patients were given courses of antibiotic therapy that exceed current guideline recommendations: 30.9% of patients with CAP were prescribed courses of 7 days or longer and 17.4% of patients with HAP were prescribed courses of 10 days or longer. All told, more than a third of antibiotic-days prescribed for pneumonia were potentially unnecessary.

Our finding that antibiotics are frequently prescribed for patients with clinical signs within reference ranges is consistent with other studies. Braykov and colleagues,^27^ for example, reviewed the medical records of 1200 patients treated with antibiotics in 6 US hospitals. The investigators reported that 30% of patients were afebrile and had white blood cell counts within the reference range on the first day of antibiotic treatment. Russell and colleagues^28^ reported that 35% of inpatients prescribed antibiotic treatment for HAP did not have new or progressive infiltrates on chest imaging. Burton and colleagues^29^ reported that 48% of patients treated for nonventilator HAP did not have compatible radiographic infiltrates, inflammatory signs, and/or respiratory signs.

Our observation that up to a third of antibiotics prescribed for possible pneumonia may be unnecessary suggests 2 potential antibiotic stewardship strategies: decrease initiation of antibiotic therapy for patients with clinical signs within reference ranges and tailor antibiotic courses to patients’ clinical trajectories. To decrease unnecessary antibiotic therapy, stewardship programs could encourage clinicians to weigh patients’ objective clinical data more heavily before prescribing antibiotics for possible pneumonia. Patients with clinical signs within reference ranges typically do not need immediate antibiotic treatment, regardless of their subjective symptoms. This concept is supported by a growing body of observational and interventional data that affirm the safety and possible benefit of awaiting confirmation of diagnosis before prescribing antibiotic treatment for clinically stable patients with possible but uncertain infections.^30,31^ Analyses of associations between time to antibiotic initiation and mortality in patients with sepsis suggest that delays in antibiotic therapy are associated with higher mortality in patients with septic shock but not those without shock.^32,33,34^ Quality improvement initiatives in which clinicians empirically prescribed antibiotic therapy only for patients with signs of possible sepsis but waited for confirmatory data before prescribing for those without sepsis suggest that this strategy is not only safe but may even confer a mortality benefit.^35,36,37,38^

Similarly, clinicians are advised to follow patients’ physiologic trajectories after starting antibiotic therapy for possible pneumonia. Clinicians should customize treatment durations to patients’ clinical trajectories rather than prospectively specifying fixed courses for all patients. At least 3 randomized trials suggest it is safe to stop antibiotic administration after as few as 3 days if patients’ clinical signs are normalizing, even if the signs are not yet completely within reference ranges.^21,22,23^ Integrating serial procalcitonin monitoring may further facilitate this strategy.^39^ Our estimate of potential excess antibiotic days is likely conservative because we only counted excess antibiotic days starting 3 days after all clinical signs had returned to the reference ranges, whereas clinical trials evaluating the safety of shorter treatment courses have permitted stopping antibiotic therapy so long as patients were improving even if some of their signs were still abnormal.

Clinical sign monitoring could potentially be the basis for a new antibiotic stewardship measurement. Rather than comparing hospitals on the average duration of antibiotic treatment prescribed for fixed diagnoses (eg, pneumonia), it might make more sense to compare average duration of antibiotic treatment beyond when clinical signs have normalized. The advantage of this approach is that it accounts for differences in clinical trajectories between patients who differ in comorbidities, infecting pathogens, and extent of infection. A measure of this nature would allow for longer courses for patients who are slow to improve and shorter courses for patients who rapidly improve. The key parameter is simply whether antibiotic therapy was continued for more than 2 days beyond when clinical signs normalized.

### Strengths and Limitations

Strengths of our study include the large sample size, the use of detailed electronic health record data to determine vital signs, inclusion of both tertiary and community hospitals, consistency of findings among hospitals, and inclusion of discharge prescriptions when calculating antibiotic durations. A further strength is our use of the indications specified by clinicians at the time of prescribing to determine why antibiotics were ordered. Using the indications specified by clinicians at the time of prescribing helps to overcome a major limitation of studies restricted to patients with either confirmed infections or discharge diagnosis codes for infections: these restrictions render these studies blind to empirical antibiotics prescribed for suspected respiratory tract infections but subsequently diagnosed with other conditions and patients with confirmed pneumonias but incomplete coding. We found that only 30% of patients who received antibiotics for pneumonia were assigned discharge diagnosis codes for pneumonia, suggesting a sustained clinical impression of pneumonia—this result is consistent with other investigations.^40^ Conversely, it is possible that clinicians may misspecify their reasons for prescribing. Our audit, however, suggested that the indications provided by clinicians largely reflected the working diagnoses recorded in their clinical notes.

Limitations of our study include the possibility that clinicians’ impressions and working diagnoses may evolve as they observe patients’ clinical courses and obtain test results. Some presentations that initially appear to be pneumonia may be due to other conditions that perhaps require longer courses of antibiotics even in the absence of ongoing symptoms (eg, endocarditis, empyema). We tried to account for this possibility by excluding patients with diagnosis codes for pyogenic complications and by conducting sensitivity analyses restricted to patients with discharge diagnosis codes for pneumonia and those with negative blood and sputum culture results. We likely underestimated the frequency of stable oxygenation on the first day of antibiotic therapy because findings of patients receiving oxygen at home or with baseline oxygen saturations below 95% were all counted as abnormal. Another limitation is the omission of radiographic imaging interpretations from our analysis. Radiographic imaging interpretations, however, are subjective, nonspecific, and correspond variably with histologic findings, and it is unlikely that a patient has pneumonia if their sole clinical sign is a radiographic opacity.^7,8,9,10^ Similarly, some patients may have had other signs or symptoms that we did not include in our analysis, such as dyspnea, cough, or chest pain. Some of these patients may have had bona fide pneumonias despite normal temperatures, respiratory rates, white blood cell counts, and oxygenation levels. In addition, our findings may not be generalizable to other hospitals and regions.

## Conclusions

In this study, we noted possible widespread overuse of antibiotics for suspected respiratory tract infections. Almost a fifth of patients started on antibiotics for possible respiratory infections had clinical signs within reference ranges and antibiotics were continued for 3 days or more beyond when clinical signs normalized in more than a third of patients. Our analysis suggests 2 potential strategies to decrease inappropriate antibiotic use for suspected respiratory infections: do not prescribe antibiotics for possible pneumonia in patients with clinical signs within reference ranges and customize treatment durations to patients’ clinical trajectories rather than prospectively specifying fixed courses for all patients. The safety and effectiveness of these strategies merit formal evaluation in future trials.

## Notes

Supplement. **eTable 1.** Clinical Signs on the First Day of Antibiotics Stratified by Hospital **eTable 2.** Duration of Antibiotics Relative to Clinical Signs Stratified by Hospital **eTable 3.** Clinical Signs on the First Day of Antibiotics **eTable 4.** Duration of Antibiotics Relative to Clinical Signs **eTable 5.** Clinical Signs on the First Day of Antibiotics in Immunocompetent vs Immunocompromised Patients **eTable 6.** Duration of Antibiotics Relative to Clinical Signs in Immunocompetent vs Immunocompromised Patients
