# Supplementary material for: Prevalence of Clinical Signs Within Reference Ranges Among Hospitalized Patients Prescribed Antibiotics for Pneumonia
Source: JAMA Netw Open. 2020 Jul 17;3(7):e2010700. doi: 10.1001/jamanetworkopen.2020.10700 (PMC7368172; doi:10.1001/jamanetworkopen.2020.10700)
Supplement: Supplement. — eTable 1. Clinical Signs on the First Day of Antibiotics Stratified by Hospital eTable 2. Duration of Antibiotics Relative to Clinical Signs Stratified by Hospital eTable 3. Clinical Signs on the First Day of Antibiotics eTable 4. Duration of Antibiotics Relative to Clinical Signs eTable 5. Clinical Signs on the First Day of Antibiotics in Immunocompetent vs Immunocompromised Patients eTable 6. Duration of Antibiotics Relative to Clinical Signs in Immunocompetent vs Immunocompromised Patients [file jamanetwopen-3-e2010700-s001.pdf]

Supplementary Online Content

Klompas M, Ochoa A, Ji W, et al; CDC Prevention Epicenters Program. Prevalence of clinical signs within reference ranges among hospitalized patients prescribed antibiotics for pneumonia. *JAMA Netw Open*. 2020;3(7):e2010700. doi:10.1001/jamanetworkopen.2020.10700

eTable 1. Clinical Signs on the First Day of Antibiotics Stratified by Hospital

eTable 2. Duration of Antibiotics Relative to Clinical Signs Stratified by Hospital

eTable 3. Clinical Signs on the First Day of Antibiotics

eTable 4. Duration of Antibiotics Relative to Clinical Signs

eTable 5. Clinical Signs on the First Day of Antibiotics in Immunocompetent vs Immunocompromised Patients

eTable 6. Duration of Antibiotics Relative to Clinical Signs in Immunocompetent vs Immunocompromised Patients

This supplementary material has been provided by the authors to give readers additional information about their work.

© 2020 Klompas M et al. *JAMA Network Open.*

eTable 1. Clinical signs on the first day of antibiotics stratified by hospital

	Academic Medical Center 1 N=3814		Academic Medical Center 2 N=5827		Community Hospital 1 N=1161		Community Hospital 2 N=1471	
	HAP N=1031	CAP N=2783	HAP N=1380	CAP N=4447	HAP N=129	CAP N=1069	HAP N=193	CAP N=1278
**Frequency of normal clinical signs on first day of antibiotics, N (%)**								
Maximum temperature >36 and <38° C	722 (70.0)	2194 (78.8)	972 (70.4)	3546 (79.7)	102 (79.1)	859 (83.2)	139 (72.0)	900 (70.4)
Median daily respiratory rate <22 breaths/minute	793 (76.9)	2234 (80.3)	1161 (84.1)	3862 (86.9)	115 (89.2)	842 (81.6)	113 (58.6)	841 (65.8)
White blood cell count >4,000 and <12,000 cells/mm^3	471 (45.7)	1513 (54.4)	667 (48.3)	2395 (53.9)	75 (58.1)	650 (63.0)	113 (58.6)	695 (54.4)
Not on supplemental oxygen	543 (52.7)	1443 (51.9)	730 (52.9)	2363 (53.1)	86 (66.7)	568 (55.0)	109 (56.5)	654 (51.2)
Oxygen saturation ≥95% without supplemental oxygen	377 (36.6)	1120 (40.2)	465 (33.7)	1752 (39.4)	49 (38.0)	404 (39.2)	64 (33.2)	441 (34.5)
Median respiratory rate <22 breaths/min and oxygen saturation ≥95% without supplemental oxygen	337 (32.7)	1040 (37.4)	446 (32.3)	1709 (38.4)	49 (38.0)	377 (36.5)	45 (23.3)	380 (29.7)
All signs normal	125 (12.1)	489 (17.6)	189 (13.7)	866 (19.5)	30 (23.3)	230 (22.3)	26 (13.5)	194 (15.2)
**Days until clinical signs normal for patients with abnormal sign(s) on the first day of antibiotics**								
Temperature, Mean (SD)	1.9 (1.6)	1.8 (1.6)	2.1 (2.2)	1.6 (1.3)	1.6 (0.9)	1.3 (0.7)	1.7 (1.6)	1.5 (0.9)
Temperature, Median (IQR)	1 (1-2)	1 (1-2)	1 (1-2)	1 (1-2)	1 (1-2)	1 (1-1)	1 (1-2)	1 (1-2)
White blood cell count, Mean (SD)	6.1 (7.0)	4.1 (4.8)	5.6 (6.5)	3.8 (4.2)	4.0 (4.0)	2.8 (2.5)	3.5 (2.9)	2.7 (2.7)
White blood cell count, Median (IQR)	3 (2-7)	2 (1-5)	3 (2-7)	2 (1-4)	2 (1-6)	2 (1-3)	3 (1-5)	2 (1-3)
Median respiratory rate, Mean (SD)	2.6 (3.5)	2.2 (2.5)	2.6 (2.7)	1.9 (1.7)	2.2 (1.7)	1.6 (1.5)	2.6 (1.9)	2.7 (2.4)
Median respiratory rate, Median (IQR)	1 (1-3)	1 (1-2)	2 (1-3)	1 (1-2)	2 (1-3)	1 (1-2)	2 (1-3)	2 (1-3)
Off supplemental oxygen, Mean (SD)	3.9 (4.6)	4.5 (4.2)	3.9 (4.0)	4.3 (4.4)	3.4 (2.8)	3.3 (3.2)	3.4 (2.5)	3.4 (2.9)
Off supplemental oxygen, Median (IQR)	2 (1-5)	3 (2-6)	3 (1-5)	3 (1-6)	3 (1-5)	2 (1-4)	3 (2-5)	3 (1-4)
Off supplemental oxygen and saturation ≥95%, Mean (SD)	6.4 (9.6)	5.0 (6.1)	5.9 (7.8)	4.8 (5.7)	3.2 (3.0)	3.3 (3.2)	3.7 (3.5)	3.4 (3.2)
Off supplemental oxygen and saturation ≥95%, Median (IQR)	3 (1-8)	3 (1-6)	3 (2-7)	3 (1-6)	2 (1-4)	2 (1-4)	3 (2-5)	2 (1-4)
Median resp rate <22 and oxygen saturation ≥95%, Mean (SD)	5.0 (5.5)	3.7 (4.4)	4.9 (5.5)	3.7 (4.6)	4.2 (3.8)	2.9 (2.1)	4.2 (6.8)	2.5 (3.4)
Median resp rate <22 and oxygen saturation ≥95%, Median (IQR)	3 (2-6)	3 (1-4)	3 (1-6)	2 (1-5)	3 (2-5)	2 (1-4)	2 (1-4)	2 (1-3)
All signs normal, Mean (SD)	7.9 (10.1)	5.6 (6.5)	7.3 (8.3)	5.3 (5.9)	4.4 (4.3)	3.7 (3.4)	4.3 (3.9)	3.9 (3.3)
All signs normal, Median (IQR)	5 (2-10)	3 (2-7)	4 (2-9)	3 (2-7)	3 (2-6)	3 (2-5)	3 (2-6)	3 (2-5)

© 2020 Klompas M et al. *JAMA Network Open.*

eTable 2. Duration of antibiotics relative to clinical signs stratified by hospital

	**Academic Medical Center 1 N=3814**		**Academic Medical Center 2 N=5827**		**Community Hospital 1 N=1161**		**Community Hospital 2 N=1471**	
	HAP N=1031	CAP N=2783	HAP N=1380	CAP N=4447	HAP N=129	CAP N=1032	HAP N=193	CAP N=1296
**Total duration of treatment**								
All patients, Mean (SD)	7.0 (6.2)	5.8 (5.7)	7.1 (8.3)	5.8 (5.8)	5.3 (3.1)	5.1 (6.3)	4.8 (3.6)	4.7 (3.6)
All patients, Median (IQR)	6 (3-9)	5 (2-8)	6 (3-8)	5 (2-7)	5 (3-7)	4 (1-7)	4 (2-7)	5 (2-6)
Patients with normal signs first day of antibiotics, Mean (SD)	5.3 (4.3)	4.9 (5.1)	5.9 (7.3)	4.8 (6.1)	5.2 (3.0)	4.0 (7.0)	4.4 (3.2)	3.9 (4.2)
Patients with normal signs first day of antibiotics, Median (IQR)	5 (2-7)	4 (1-6)	5 (2-7)	4 (1-6)	5 (3-7)	2 (1-5)	4 (2-6)	3 (1-5)
Patients with abnormal signs first day of antibiotics, Mean (SD)	7.2 (6.4)	6.1 (5.8)	7.2 (8.4)	6.0 (5.7)	5.3 (3.1)	5.4 (6.1)	4.8 (3.7)	4.8 (3.5)
Patients with abnormal signs first day of antibiotics, Median (IQR)	6 (3-9)	5 (2-8)	6 (3-8)	5 (2-8)	5 (3-7)	5 (2-7)	5 (2-7)	5 (2-6)
**Duration of treatment after all signs normal**								
All patients, Mean (SD)	3.3 (5.4)	2.5 (4.3)	3.2 (7.1)	2.6 (4.7)	3.0 (3.7)	2.8 (5.9)	2.2 (3.8)	2.0 (3.0)
All patients, Median (IQR)	0 (0-5)	1 (0-4)	1 (0-5)	1 (0-4)	2 (0-5)	1 (0-4)	0 (0-4)	1 (0-3)
Patients with abnormal signs first day of antibiotics, Mean (SD)	2.5 (4.3)	2.3 (4.3)	2.5 (6.9)	2.3 (4.4)	2.0 (2.9)	2.6 (5.7)	1.7 (2.8)	1.8 (2.7)
Patients with abnormal signs first day of antibiotics, Median (IQR)	0 (0-4)	0 (0-4)	1 (0-4)	1 (0-4)	0 (0-4)	1 (0-4)	0 (0-3)	0 (0-3)
**Counts of patients with extended courses of antibiotics**								
≥3 days antibiotics beyond last day of abnormal signs, Count (%)	410 (39.8)	939 (33.7)	519 (37.6)	1590 (35.8)	58 (45.0)	373 (36.1)	63 (32.6)	420 (32.9)
≥5 days antibiotics beyond last day of abnormal signs, Count (%)	285 (27.6)	587 (21.1)	351 (25.4)	902 (20.3)	38 (29.5)	206 (20.0)	34 (17.6)	192 (15.0)

© 2020 Klompas M et al. *JAMA Network Open.*

eTable 3. Clinical signs on the first day of antibiotics (primary analysis vs sensitivity analyses restricted to patients with discharge diagnosis codes for pneumonia and patients with negative blood and sputum cultures)

	**All Patients**		**Patients with Discharge Codes for Pneumonia**		**Patients with Negative Blood and Sputum Cultures**	
	**Hospital-Acquired Pneumonia (N=2,733)**	**Community-Acquired Pneumonia (N=9,540)**	**Hospital-Acquired Pneumonia (N=530)**	**Community-Acquired Pneumonia (N=3,228)**	**Hospital-Acquired Pneumonia (N=582)**	**Community-Acquired Pneumonia (N=1,994)**
**Frequency of normal signs on the first day of antibiotics**						
Maximum temperature >36 and <38° C	1935 (70.8)	7499 (78.6)	367 (69.3)	2374 (73.5)	298 (51.2)	1333 (66.9)
Median daily respiratory rate ≤22 breaths/minute	2182 (79.8)	7779 (81.5)	449 (84.7)	2596 (80.4)	434 (74.6)	1520 (76.2)
Maximum WBC count >4,000 and <12,000 cells/mm^3	1326 (48.5)	5253 (55.1)	261 (49.3)	1646 (51.0)	259 (44.5)	928 (46.5)
Not on supplemental oxygen	1468 (53.7)	5028 (52.7)	298 (56.2)	1686 (52.2)	255 (43.8)	892 (44.7)
Oxygen saturation ≥95% without supplemental oxygen	955 (34.9)	3717 (39.0)	197 (37.2)	1217 (37.7)	153 (26.3)	614 (30.8)
Median respiratory rate <22 breaths/minute and oxygen saturation ≥95% without supplemental oxygen	877 (32.1)	3506 (36.8)	191 (36.0)	1150 (35.6)	134 (23.0)	565 (28.3)
All signs normal	370 (13.5)	1779 (18.7)	84 (15.9)	526 (16.3)	41 (7.0)	196 (9.8)
**Days until clinical signs normal for patients with abnormal sign(s) on the first day of antibiotics**						
Temperature, Mean (SD)	2.0 (1.9)	1.6 (1.3)	2.3 (2.6)	1.6 (1.2)	2.2 (2.0)	1.7 (1.3)
Temperature, Median (IQR)	1 (1-2)	1 (1-2)	1 (1-3)	1 (1-2)	1 (1-3)	1 (1-2)
White blood cell count, Mean (SD)	5.7 (6.5)	3.7 (4.1)	5.2 (5.8)	3.3 (3.2)	6.1 (7.7)	4.3 (4.5)
White blood cell count, Median (IQR)	3 (2-7)	2 (1-4)	3 (2-6)	2 (1-4)	3 (2-7)	3 (1-5)
Median respiratory rate, Mean (SD)	2.6 (3.0)	2.2 (2.2)	2.7 (3.1)	2.2 (2.0)	2.5 (2.2)	2.6 (2.4)
Median respiratory rate, Median (IQR)	2 (1-3)	1 (1-3)	2 (1-3)	1 (1-3)	2 (1-3)	2 (1-3)
Off supplemental oxygen, Mean (SD)	3.8 (4.1)	4.1 (4.1)	4.1 (4.1)	4.0 (3.7)	3.3 (3.7)	4.4 (4.6)
Off supplemental oxygen, Median (IQR)	2 (1-5)	3 (1-6)	2 (1-5)	3 (1-6)	2 (1-4)	3 (1-6)
Off supplemental oxygen and saturation ≥95%, Mean (SD)	5.8 (8.2)	4.5 (5.4)	4.9 (5.8)	4.4 (5.0)	7.5 (10.8)	5.6 (6.5)
Off supplemental oxygen and saturation ≥95%Median (IQR)	3 (1-7)	3 (1-6)	3 (1-6)	3 (1-6)	4 (2-9)	4 (2-7)
Median resp rate <22 and O_2 saturation ≥95%, Mean (SD)	4.9 (5.5)	3.5 (4.3)	4.4 (5.5)	3.1 (3.1)	4.9 (5.5)	3.5 (3.2)
Median resp rate <22 and O_2 saturation ≥95%, Median (IQR)	3 (1-6)	2 (1-4)	3 (1-5)	2 (1-4)	4 (1-6)	3 (1-5)
All signs normal, Mean (SD)	7.2 (8.8)	5.0 (5.7)	6.1 (6.4)	4.8 (5.1)	9.1 (11.4)	6.2 (6.5)
All signs normal, Median (IQR)	4 (2-9)	3 (2-6)	4 (2-8)	3 (2-6)	5 (3-11)	4 (2-8)

© 2020 Klompas M et al. *JAMA Network Open.*

eTable 4. Duration of antibiotics relative to clinical signs (primary analysis vs sensitivity analysis restricted to patients with discharge diagnosis codes for pneumonia and patients with negative blood and sputum cultures)

	All Patients		Patients with Discharge Codes for Pneumonia		Patients with Negative Sputum and Blood Cultures	
	Hospital-Acquired Pneumonia (N=2,733)	Community-Acquired Pneumonia (N=9,540)	Hospital-Acquired Pneumonia (N=530)	Community-Acquired Pneumonia (N=3,228)	Hospital-Acquired Pneumonia (N=582)	Community-Acquired Pneumonia (N=1,994)
**Total duration of treatment in days**						
All patients, Mean (SD)	6.8 (7.2)	5.6 (5.6)	7.5 (5.4)	6.4 (5.3)	8.0 (7.1)	7.3 (5.6)
All patients, Median (IQR)	5 (3-8)	5 (2-7)	7 (4-9)	6 (3-8)	7 (4-9)	6 (4-9)
Patients with normal signs on first day of antibiotics, Mean (SD)	6.2 (9.6)	4.7 (5.6)	6.1 (4.7)	5.3 (5.4)	6.9 (5.1)	6.0 (4.8)
Patients with normal signs on first day of antibiotics, Median (IQR)	5 (3-7)	4 (1-6)	5 (3-8)	5 (2-7)	6 (4-8)	5 (2-8)
Patients with abnormal signs on first day of antibiotics, Mean (SD)	6.9 (6.4)	5.9 (5.6)	7.8 (5.5)	6.6 (5.3)	8.0 (7.2)	7.4 (5.7)
Patients with abnormal signs on first day of antibiotics, Median (IQR)	6 (3-8)	5 (2-7)	7 (5-9)	6 (3-8)	7 (4-10)	7 (4-9)
**Duration of treatment beyond the last day of abnormal signs**						
All patients, Mean (SD)	3.2 (6.2)	2.5 (4.5)	3.7 (5.0)	3.1 (4.4)	2.8 (4.7)	3.0 (4.6)
All patients, Median (IQR)	1 (0-5)	1 (0-4)	2 (0-5)	2 (0-5)	0 (0-4)	1 (0-4)
Patients with abnormal signs on first day of antibiotics, Mean (SD)	2.4 (5.7)	2.3 (4.3)	3.0 (4.2)	2.9 (4.4)	2.6 (4.6)	2.8 (4.5)
Patients with abnormal signs on first day of antibiotics, Median (IQR)	0 (0-4)	0 (0-4)	2 (0-5)	2 (0-4)	0 (0-4)	1 (0-4)
**Counts of patients with extended courses of antibiotics**						
≥3 days of antibiotics beyond the last day of abnormal signs, Count (%)	1050 (38.4)	3322 (34.8)	259 (48.9)	1436 (44.5)	219 (37.6)	784 (39.3)
≥5 days of antibiotics beyond the last day of abnormal signs, Count (%)	708 (25.9)	1887 (19.8)	168 (31.7)	844 (26.2)	129 (22.2)	485 (24.3)

© 2020 Klompas M et al. *JAMA Network Open.*

eTable 5. Clinical signs on the first day of antibiotics in immunocompetent vs immunocompromised patients

	**Immunocompetent Patients**		**Immunocompromised Patients**	
	**Hospital-Acquired Pneumonia (N=2,004)**	**Community-Acquired Pneumonia (N=7,745)**	**Hospital-Acquired Pneumonia (N=729)**	**Community-Acquired Pneumonia (N=1,795)**
**Frequency of normal signs on the first day of antibiotics**				
Daily maximum temperature >36 and <38° C	1480 (73.9)	6174 (79.7)	455 (62.4)	1325 (73.8)
Median daily respiratory rate ≤22 breaths/minute	1585 (79.1)	6257 (80.8)	597 (81.9)	1522 (84.8)
Daily maximum WBC count >4,000 and <12,000 cells/mm^3	1056 (52.7)	4446 (57.4)	270 (37.0)	807 (45.0)
Not on supplemental oxygen	1041 (52.0)	4053 (52.3)	427 (58.6)	975 (54.3)
Oxygen saturation ≥95% without supplemental oxygen	658 (32.8)	2977 (38.4)	297 (40.7)	740 (41.2)
Median respiratory rate <22 breaths/minute and oxygen saturation ≥95% without supplemental oxygen	593 (29.6)	2789 (36.0)	284 (39.0)	717 (39.9)
All signs normal	297 (14.8)	1525 (19.7)	73 (10.0)	254 (14.2)
**Days until clinical signs normal for patients with abnormal sign(s) on the first day of antibiotics**				
Temperature, Mean (SD)	1.8 (1.6)	1.5 (1.2)	2.3 (2.4)	2.0 (1.6)
Temperature, Median (IQR)	1 (1-2)	1 (1-2)	1 (1-3)	1 (1-2)
White blood cell count, Mean (SD)	4.9 (5.4)	3.2 (3.4)	7.3 (8.1)	5.3 (5.6)
White blood cell count, Median (IQR)	3 (1-6)	2 (1-4)	4 (2-9)	3 (2-7)
Median respiratory rate, Mean (SD)	2.6 (3.1)	2.1 (2.2)	2.8 (2.7)	2.3 (2.1)
Median respiratory rate, Median (IQR)	2 (1-3)	1 (1-2)	2 (1-3)	2 (1-3)
Off supplemental oxygen, Mean (SD)	3.6 (4.0)	4.0 (4.0)	4.5 (4.4)	5.0 (4.5)
Off supplemental oxygen, Median (IQR)	2 (1-5)	3 (1-5)	3 (1-6)	4 (2-7)
Off supplemental oxygen and saturation ≥95%, Mean (SD)	5.8 (8.5)	4.3 (5.2)	5.9 (7.2)	5.6 (6.1)
Off supplemental oxygen and saturation ≥95%, Median (IQR)	3 (1-7)	3 (1-5)	3 (2-8)	4 (2-7)
Median resp rate <22 and oxygen saturation ≥95%, Mean (SD)	4.4 (4.9)	3.2 (3.8)	6.0 (6.5)	4.5 (5.6)
Median resp rate <22 and oxygen saturation ≥95%, Median (IQR)	3 (1-5)	2 (1-4)	4 (2-8)	3 (2-5)
All signs normal, Mean (SD)	6.8 (8.5)	4.6 (5.1)	8.3 (9.3)	6.7 (7.1)
All signs normal, Median (IQR)	4 (2-8)	3 (2-6)	5 (2-11)	5 (2-8)

© 2020 Klompas M et al. *JAMA Network Open.*

eTable 6. Duration of antibiotics relative to clinical signs in immunocompetent vs immunocompromised patients

	**Immunocompetent Patients**		**Immunocompromised Patients**	
	**Hospital-Acquired Pneumonia (N=2,004)**	**Community-Acquired Pneumonia (N=7,745)**	**Hospital-Acquired Pneumonia (N=729)**	**Community-Acquired Pneumonia (N=1,795)**
**Total duration of treatment in days**				
All patients, Mean (SD)	6.2 (6.3)	5.2 (5.1)	8.4 (9.0)	7.4 (7.1)
All patients, Median (IQR)	5 (3-8)	5 (2-7)	6 (3-10)	6 (3-9)
Patients with normal signs on first day of antibiotics, Mean (SD)	5.1 (4.3)	4.2 (5.1)	7.3 (9.9)	6.9 (8.6)
Patients with normal signs on first day of antibiotics, Median (IQR)	5 (2-7)	3 (1-6)	6 (3-8)	5 (2-8)
Patients with abnormal signs on first day of antibiotics, Mean (SD)	6.4 (6.5)	5.4 (5.1)	8.5 (8.8)	7.4 (6.8)
Patients with abnormal signs on first day of antibiotics, Median (IQR)	5 (3-8)	5 (2-7)	7 (3-10)	6 (3-9)
**Duration of treatment beyond the last day of abnormal signs**				
All patients, Mean (SD)	3.0 (6.1)	2.4 (4.4)	3.6 (6.3)	2.9 (5.1)
All patients, Median (IQR)	1 (0-5)	1 (0-4)	0 (0-5)	1 (0-4)
Patients with abnormal signs on first day of antibiotics, Mean (SD)	2.2 (5.6)	2.2 (4.3)	3.0 (5.9)	2.7 (4.5)
Patients with abnormal signs on first day of antibiotics, Median (IQR)	0 (0-4)	0 (0-3)	0 (0-4)	0 (0-4)
**Counts of patients with extended courses of antibiotics**				
≥3 days of antibiotics beyond the last day of abnormal signs, Count (%)	771 (38.5)	2672 (34.5)	279 (38.3)	650 (36.2)
≥5 days of antibiotics beyond the last day of abnormal signs, Count (%)	612 (30.5)	1684 (21.7)	200 (27.4)	434 (24.2)

© 2020 Klompas M et al. *JAMA Network Open.*
